# Quantitative Analysis of CT Images in Patients with Pyrrolizidine Alkaloid-Induced Sinusoidal Obstruction Syndrome

**DOI:** 10.1038/s41598-019-38669-6

**Published:** 2019-02-18

**Authors:** Chao Wang, Xingwang Wu, Wentao Xie, Xiaofei Ren, Weiping Zhang, Jianming Xu

**Affiliations:** 1 Department of Gastroenterology, The First Affiliated Hospital of Anhui Medical University, Hefei, China; 2 Department of Radiology, The First Affiliated Hospital of Anhui Medical University, Hefei, China; 3 Department of Vascular Surgery, The First Affiliated Hospital of Anhui Medical University, Hefei, China

## Abstract

This study evaluated hepatic lesion volumes on contrast-enhanced computed tomography (CT) images in patients with pyrrolizidine alkaloid-induced sinusoidal obstruction syndrome (PA-SOS) and the association of lesion volume with the clinical severity and prognosis of the disease. Twenty-five patients with PA-SOS were included in this study, and all patients were subjected to a complete CT imaging series. The imaging results were quantitatively analyzed by a threshold-based region growing algorithm. The liver volumes and hepatic lesion volumes of the patients were estimated. Based on clinical presentations, PA-SOS was classified into three categories: mild, moderate and severe. The associations of hepatic lesion volumes with liver function test parameters and the clinical severity and prognosis of the disease were analyzed. Based on estimations using the threshold-based region growing algorithm, hepatic lesion volumes in patients with mild PA-SOS were significantly lower than those in patients with moderate and severe PA-SOS (P < 0.05). The ratio of hepatic lesion volume to liver volume (Ratio) varied based on alanine aminotransferase (ALT), aspartate aminotransferase (AST) and serum total bilirubine levels; clinical severity; and disease prognosis, and the differences were statistically significant (P < 0.05). In conclusion, the threshold-based region growing algorithm can be employed to quantitatively analyze enhanced CT images of PA-SOS patients. And the ratio of hepatic lesion volume to liver volume in patients with PA-SOS is associated with a more serious clinical course and a poorer outcome.

## Introduction

Sinusoidal obstruction syndrome (SOS), also known as veno-occlusive disease (VOD), is a potentially life-threatening disease. It is clinically characterized by hepatomegaly, ascites and jaundice due to damage to sinusoidal endothelial cells and congestion of the hepatic sinusoids^1,2^. One of the major etiologies of SOS is the intake of pyrrolizidine alkaloid (PA)-containing products, such as *Gynura segetum* (Tusanqi), a traditional Chinese herbal medicine^3–5^. The clinical presentations of PA-SOS are nonspecific, but characteristic changes of sinusoidal dilation and congestion can be observed in the liver biopsies of patients with the disease^2,4–6^. The diagnosis of PA-induced SOS (PA-SOS) mostly depends on computed tomography (CT) imaging and a history of PA consumption^7^ because liver biopsy is invasive and cannot be performed in patients with extensive ascites. Furthermore, several studies have shown that the grade of hepatic changes in patients with SOS, which is determined according to heterogeneous hypoattenuation areas on enhanced CT images, is associated with the clinical severity of the disease^8,9^. However, the methods used to classify the severity of the disease based on imaging findings mainly include visual assessment and lack objective and quantitative parameters. Thus, a method to quantitatively measure hepatic lesions in patients with SOS is necessary. A threshold-based region growing algorithm can be applied to quantitatively analyze CT images^10,11^. Therefore, the objectives of this study were to establish a method for quantitative measurement of hepatic lesion volumes on CT images and to investigate the relationship between the measurements and the clinical severity and prognosis of the disease.

## Results

### The measurements based on the threshold-based region growing algorithm and the characteristics of the patients

Table 1 summarizes the liver volume, hepatic lesion volume, and the ratio of the two volumes (Ratio) obtained by the threshold-based region growing algorithm during the initial CT examinations. The average liver volume in the group of patients with PA-SOS was 1.727 ± 0.398 L. Heterogeneous low-density areas (ROI _lesion_) were present on the CT images of 25 patients with PA-SOS, and the volume of the areas was 1.015 ± 0.355 L. In addition, the clinical features of these patients and laboratory test results were also evaluated (Table 2). Ascites was the most common feature in PA-SOS, while hepatomegaly and jaundice were evident in most patients. Liver function tests during the same period showed that biomarkers of liver damage (ALT and AST) and cholestasis (ALP and γ-GT) and serum total bilirubin exceeded the upper limits of their respective normal ranges, while erythrocyte, leukocyte and platelet counts were within normal ranges, as was creatinine (Cr) in kidney function tests.

**Table 1:** Summary of Patients with PA-SOS and Measurements.

Patient	Sex	Age (y)	ROI _liver_ (L)	ROI _lesion_ (L)	Ratio
Case 1	F	69	1.306	0.833	0.6378
Case 2	F	44	1.166	0.498	0.4271
Case 3	M	74	1.342	0.949	0.7072
Case 4	F	70	1.289	0.956	0.7417
Case 5	F	80	2.018	0.798	0.3835
Case 6	F	58	1.921	1.426	0.7423
Case 7	F	59	1.545	0.538	0.3482
Case 8	M	71	2.069	1.453	0.7023
Case 9	M	50	2.136	1.300	0.6086
Case 10	F	43	1.950	0.999	0.5123
Case 11	F	66	1.857	0.739	0.3980
Case 12	F	67	1.032	0.671	0.6502
Case 13	M	42	1.584	1.015	0.6408
Case 14	M	73	1.413	0.762	0.5393
Case 15	M	74	2.200	1.412	0.6418
Case 16	M	65	1.850	1.295	0.7000
Case 17	M	47	1.808	1.518	0.8396
Case 18	F	66	1.389	1.021	0.7351
Case 19	M	49	2.335	1.650	0.7066
Case 20	M	29	2.617	1.476	0.5640
Case 21	F	62	1.769	1.052	0.5947
Case 22	M	66	1.722	1.242	0.7213
Case 23	F	70	1.976	0.545	0.2758
Case 24	M	71	1.281	0.767	0.5988
Case 25	F	62	1.534	0.465	0.3031
Average		61.08 ± 12.70	1.727 ± 0.398	1.015 ± 0.355	0.5888 ± 0.1535

**Table 2:** Clinical Manifestations of the Patients and Laboratory Test Results.

Clinical manifestation	Patients with PA-SOS
	n/N (%)
hepatomegaly	18/25 (72.00%)
ascites	25/25 (100%)
jaundice	16/25 (64%)
Laboratory tests^*^	Mean ± SD
erythrocytes	4.61 ± 0.49
HGB	135.92 ± 16.16
leukocytes	5.94 ± 1.63
PLT	95.52 ± 37.07
ALT	103.56 ± 123.27
AST	155.56 ± 166.00
ALP	153.04 ± 67.00
γ-GT	139.44 ± 95.24
total bilirubin	49.46 ± 33.29
albumin	34.02 ± 4.34
PT	17.28 ± 3.42
INR	1.45 ± 0.34
Cr	83.68 ± 37.72
BUN	7.57 ± 3.91

*Normal ranges for the laboratory test results: erythrocytes: 3.0–5.5 × 10^12^/L, hemoglobin: 110–160 g/L, leukocytes: 4–10 × 10^9^/L, platelets: 100–300 × 10^9^/L, alanine aminotransferase (ALT): 5–35 U/L, aspartate aminotransferase (AST): 8–40 U/L, alkaline phosphatase (ALP) 40–150 U/L, albumin: 35–55 g/L, total bilirubin: 5.1–19 μmol/L, γ-glutamyl-transferase (γ-GT) 7–32 U/L, prothrombin time (PT): 11–16 s, creatinine: 44–106 μmol/L, blood urea nitrogen (BUN): 1.8–7.1 mmol/L.

### Relationship between quantitative measurements and clinical severity in patients with PA-SOS

The ROI _lesion_ volumes were compared and correlated with clinical severity. The volume of the ROI _lesion_ was significantly lower in patients with mild PA-SOS than it was in patients with moderate and severe PA-SOS (P < 0.05). Additionally, the Ratio varied according to the clinical severity of the disease, and the difference was statistically significant (P < 0.05) (Table 3).

**Table 3:** Comparison of Liver Volumes, Hepatic Lesion Volumes and Ratios with Clinical Severity in Patients of PA-SOS.

	Mild (n = 6)	Moderate (n = 10)	Severe (n = 9)	F value	P value
ROI _liver_ (L)	1.824 ± 0.232	1.743 ± 0.552	1.645 ± 0.289	0.357	>0.05
ROI _lesion_ (L)	0.681 ± 0.202	1.067 ± 0.381	1.015 ± 0.355	4.986	<0.05*
Ratio	0.3701 ± 0.0837	0.6070 ± 0.0742	0.7143 ± 0.0781	35.638	<0.05

^*^The volume of ROI _lesion_ in patients with mild PA-SOS was significantly lower than that in patients with moderate and severe PA-SOS.

In addition, liver functional test parameters were also compared with the severity of the disease. The results showed that the alanine aminotransferase (ALT), aspartate aminotransferase (AST) and serum total bilirubin levels varied according to the severity of the disease (Table 4).

**Table 4:** Comparison of the Parameter of Liver Functional Test with Clinical Severity in Patients of PA-SOS.

	Mild (n = 6)	Moderate (n = 10)	Severe (n = 9)	F value	P value
ALT (U/L)	42.50 ± 33.71	58.20 ± 35.98	194.67 ± 169.44	5.239	<0.05
AST (U/L)	58.33 ± 35.86	91.40 ± 47.01	291.67 ± 215.17	7.337	<0.05
ALP (U/L)	124.17 ± 31.13	165.40 ± 93.10	158.56 ± 47.51	0.742	>0.05
γ-GT (U/L)	92.33 ± 38.91	160.50 ± 135.75	147.44 ± 56.93	1.011	>0.05
Total bilirubin (μmol/L)	19.93 ± 10.54	43.23 ± 14.49	76.06 ± 38.98	9.033	<0.05
Albumin (g/L)	34.35 ± 5.45	34.52 ± 3.44	33.23 ± 4.84	0.217	>0.05

The correlations between the Ratio and the ALT, AST and serum total bilirubin levels were determined by the Pearson correlation test. Significant associations were observed between the Ratio and ALT level (ρ = 0.400, P < 0.05), AST level (ρ = 0.519, P < 0.05) and serum total bilirubin level (ρ = 0.543, P < 0.05).

### Relationships between quantitative measurements and the prognosis of patients with PA-SOS

According to the follow-up results for the patients with PA-SOS, six patients recovered, nine patients had cirrhosis, and six patients died. Compared with the CT imaging measurements, the Ratio was associated with the incidence of death or cirrhosis (P < 0.05, Table 5).

**Table 5:** Comparison of Liver Volumes, Hepatic Lesion Volumes and Ratios with Clinical Outcomes in Patients with PA-SOS.

	Recovery (n = 10)	Cirrhosis (n = 9)	Death (n = 6)	F value	P value
ROI _liver_ (L)	1.938 ± 0.403	1.558 ± 0.364	1.629 ± 0.322	2.738	>0.05
ROI _lesion_ (L)	0.939 ± 0.428	1.006 ± 0.300	1.156 ± 0.309	0.683	>0.05
Ratio	0.4732 ± 0.1512	0.6398 ± 0.0322	0.7050 ± 0.0969	8.000	<0.05

## Discussion

SOS is mostly observed after hematopoietic stem cell transplantation (HSCT) and is associated with risk factors such as high intensity of the conditioning regimen^2,12,13^. SOS induced by PA-containing herbal medicine has been increasingly reported in recent years. Ge Lin *et al*. reported that more than 8,000 cases of PA-SOS had been documented worldwide by 2011, at least 51 of which were attributed to *Gynura segetum* (Tusanqi) intake^4^. The diagnosis of the disease depends mostly on the Seattle criteria and Baltimore criteria^14,15^, which are more applicable for the diagnosis of HSCT-related SOS. However, the diagnosis of PA-SOS should consider the intake of PA-containing products and typical CT imaging findings^7^. Patchy enhancement and heterogeneous hypoattenuation are the most informative features on contrast CT images. One study reported a sensitivity of 92.96% and a specificity of 92.79% for patchy enhancement and a sensitivity of 100% and a specificity of 95.05% for heterogeneous hypoattenuation, illustrating diagnostic efficacy compared with the Seattle criteria^8^. This finding suggests that CT imaging is important for the diagnosis of PA-SOS. However, several studies have reported that heterogeneous hypointensity was also found on most magnetic resonance imaging (MRI) images of patients with PA-SOS^9,16^. Unfortunately, only a small proportion of the enrolled patients received MRI examinations. Therefore, the MRI images of the patients with PA-SOS were not evaluated in this study.

Furthermore, the grade of hepatic lesions on CT imaging and clinical severity have been reported to be correlated. Xuefeng Kan *et al*. reported that heterogeneous hypoattenuation was obviously related to the incidence and severity of PA-SOS^8^, while Hua Zhou *et al*. found that the grade of hepatic lesions in patients with PA-SOS was correlated with clinical severity^9^. Additionally, in another study, heterogeneous hypointensity was also found on most MRI images of patients with PA-SOS and was significantly associated with ALT (ρ = 0.409, P = 0.02) and AST levels (ρ = 0.509, P = 0.003)^16^. However, the extent of heterogeneous hypoattenuation on CT images or heterogeneous hypointensity on MRI images was visually assessed and lacked objective and quantitative indicators. A threshold-based region growing algorithm can be used to extract ROIs such that ROI volumes in PA-SOS patients can be calculated. An advantage of this study is that the areas of hepatic lesions on contrast CT images of patients with PA-SOS can be quantitatively analyzed by the threshold-based region growing algorithm. Then, the measurements were compared and correlated with liver function test parameters, clinical severity and disease prognosis. The ROI _lesion_ volumes in patients with mild disease were significantly lower than those in patients with moderate or severe disease. Furthermore, a significant difference between the Ratio and clinical severity was identified based on the CT images of patients with PA-SOS. Greater clinical severity corresponded to a higher Ratio. Simultaneously, liver function test results were compared and correlated with clinical severity in patients with PA-SOS. The results showed significant associations between clinical severity and ALT and AST levels, which is consistent with the findings in a previous study. However, in our study, we found that the serum total bilirubin level also varied according to the severity of the disease. We also determined the correlations between the Ratio and the ALT, AST and serum total bilirubin levels and found that these levels were significantly associated with the Ratio. Another finding in our study was a significant difference between the Ratio and clinical outcomes, suggesting that quantitative measurement of ROI lesion volumes in patients with PA-SOS by using the threshold-based region growing algorithm can be used to predict their prognoses.

Quantitatively measuring the extent of hepatic lesions in patients with PA-SOS may assist in assessing the severity of the disease and developing intervention strategies. Because their clinical severities and clinical interventions varied, the patients with SOS had significantly different outcomes. Nelson Chao reported that patients with mild SOS have a tendency to recover by themselves, while patients with moderate and severe SOS may die or progress to cirrhosis^17^. It has also been suggested that SOS treatment be modulated according to the severity of the disease. Defibrotide has been considered the only effective treatment for the disease. Paul G. Richardson *et al*. reported that patients with SOS treated with defibrotide experienced significant improvements in their 100-day survival and remission rates^18^. However, due to cost and other factors, the strategy cannot be universally applied and is used only in patients with severe disease and in the early stages of the disease. Moreover, quantitative analysis of CT images can lead to preliminary indications of disease severity, which may facilitate treatment during the clinical course of the disease.

A limitation of the study may be that pathological evidence was not available due to ascites and coagulation disorders in the patients with PA-SOS. Therefore, a quantitative histological analysis could not be performed. However, the obstructed areas manifested as heterogeneous hypoattenuation or heterogeneous low-density areas because of the delay in the uptake and obstruction of the contrast agent. Thus, hepatic lesion areas could be detected on CT images. Nevertheless, more research should be performed to correlate histological changes with CT imaging findings.

Another limitation of the study may be the relatively small number of subjects. However, our work was focused on establishing a novel method for estimating hepatic lesion volume and investigating its applicability to clinical practice. In particular, the method can provide objective CT imaging evidence for treatment during the clinical course and follow-up. Of course, the method must be tested and evaluated in a larger patient sample.

## Conclusion

In summary, a threshold-based region growing algorithm can be employed to quantitatively analyze contrast CT images of patients with PA-SOS. A higher ratio of ROI _lesion_ to ROI _liver_ volumes on contrast CT images in patients with PA-SOS corresponds to greater clinical severity and a worse prognosis. Quantitative measurement of heterogeneous low-density areas and the ratio of ROI _lesion_ to ROI _liver_ volumes are highly reliable for assessing the severity and prognosis of PA-SOS.

## Methods

All methods were carried out in accordance with relevant regulations and guidelines.

### Patients

**Figure 1:**
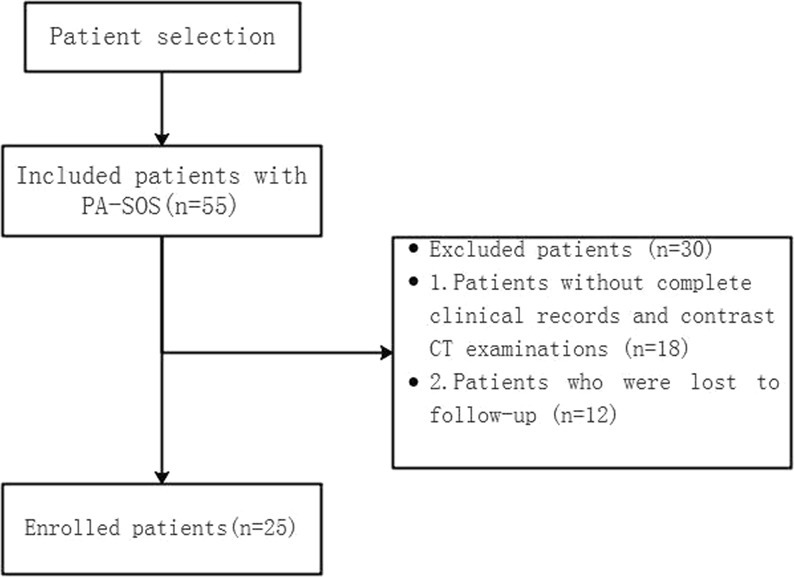
Flowchart for patient selection.

Fifty-five consecutive patients diagnosed with PA-SOS were admitted to our hospital between December 1, 2009, and July 31, 2017. Patients with other liver diseases or incomplete clinical records and/or contrast CT examinations and patients who were lost to follow-up were excluded. Finally, 25 patients with PA-SOS, 12 males and 13 females (age range, 29–80 years; mean age, 61.08 ± 12.70 years), were enrolled (Fig. 1).

The criteria for the diagnosis of PA-SOS were generally based on the Seattle and Baltimore clinical criteria with modifications^7,19^ including a history of PA-containing drug intake and fulfillment of three of the following five items: (1) abdominal distention, hepatomegaly and ascites; (2) an increased serum total bilirubin level; (3) liver biopsy revealing obstruction and extension of the hepatic sinusoids in zone 3 of the hepatic acinus; (4) typical findings on enhanced CT examinations; and (5) detection of pyrrole protein adducts in serum.

Furthermore, the severity of the disease based on the classification of the clinical course used for retrospective assessment was as follows^12,20^: (1) mild SOS, the disease may have been clinically obvious but was resolved after cessation of exposure or after receiving simple drug treatment; (2) moderate SOS, symptoms required supportive treatment, such as diuretics or pain medication; and (3) severe SOS, the disease required treatment but did not resolve after 100 days of treatment and was complicated with multiple organ dysfunction syndrome or led to mortality.

Patients with PA-SOS were followed up by one of the following three methods: outpatient visits, inpatient visits or telephone calls. Follow-up evaluations were performed via outpatient visits for one patient, inpatient visits for 6 patients, and telephone calls for 18 patients. The patients were followed up every 3 to 6 months after discharge from the hospital. The end point of the follow-up was the date of death or January 31, 2018; the follow-up time ranged from 4 to 65 months, and the average follow-up time was 30.68 ± 16.98 months. The clinical outcome was classified as follows: recovery, liver cirrhosis or death.

### Imaging technique

All patients were examined by three-phase liver CT (64-multidetector CT scanner LightSpeed, GE Healthcare, Milwaukee, WI, USA) after an intravenous injection of contrast media (100 mL of Omnipaque, GE Healthcare Co. Ltd, China; 300 mgI/mL at a rate of 3.0 mL/s) at 25–30, 55–70 and 90–120 seconds, respectively. Timing for arterial phase imaging was determined by using a bolus-tracking technique or a test-bolus injection technique. The scanning parameters were 120 kVp, 500 mA, 5-mm section thickness and 1.375 pitch.

### ROI definition

Patchy liver enhancement and heterogeneous hypoattenuation have been reported as the most typical manifestations of SOS on CT images. Because SOS causes sinusoid congestion and therefore delays contrast agent inflow, hepatic lesion areas appeared darker on contrast CT images and exhibited heterogeneous hypoattenuation or heterogeneous low density^21^. The imaging changes mentioned above were most obvious at the hepatic venous phase on contrast CT^22^. Therefore, CT images at the hepatic venous phase were analyzed for quantitative measurements. Regions of interest (ROIs) were defined for the hypoattenuation area, enhancement area and liver.

**Figure 2:**
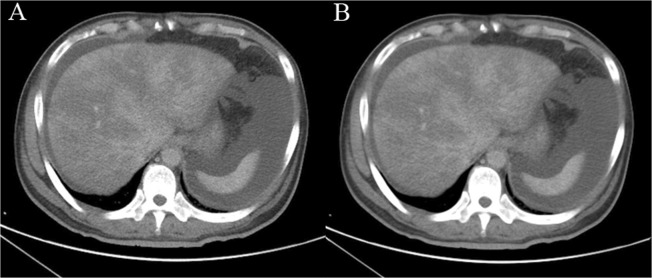
(**A**) 49-year-old man was diagnosed with PA-SOS and underwent contrast-enhanced CT. CT images were denoised using the anisotropic diffusion method; (**A**) original and (**B**) denoised.

Liver extraction and measurement without denoising are difficult because the intensity distribution of the liver is irregular due to noise^23^. The anisotropic diffusion method can reduce the effect of noise while preserving the boundaries and fine details of organs and tissues on a CT image. The parameters of the anisotropic diffusion method were as follows: iterations = 4 and time step = 0.125. CT images of patients with PA-SOS were denoised through an anisotropic diffusion filter^24^ (Fig. 2).

### Threshold-based region growing method

The threshold-based region growing algorithm is an image segmentation method applied with region growing based on the Hounsfield unit (HU) of the ROI and can be used to extract the ROI^24^. To determine the ROI of the liver (ROI _liver_), the approximate range of the liver was first manually delineated, and the threshold of the ROI _liver_ was then obtained by Analyze 12.0 software (Mayo Clinic College of Medicine, Rochester, Minnesota). Based on the threshold of the ROI _liver_, region growing was performed to extract the ROI _liver_, and then the ROI _liver_ volume was calculated.

**Figure 3:**
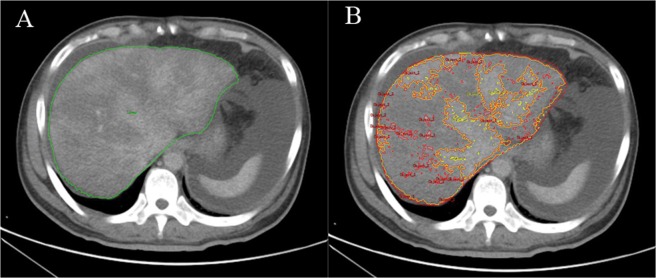
ROIs were extracted by the threshold-based region growing method (ROI_liver,_ ROI_lesion_ and ROI_enhancement_ are represented by the green line, yellow line and red line, respectively): The volumes for (**A**) ROI_liver_ and (**B**) ROI_lesion_ were 2334821.84 mm^3^ (2.335 L) and 1650085.09 mm^3^ (1.650 L), respectively.

When employing the method to analyze the ROI of a lesion (ROI_lesion_), the threshold of the ROI of low-density areas (ROI_low density_) and the ROI of the enhancement areas (ROI_enhancement_) were initially obtained. For the ROI _low density_, the threshold was obtained as [a, b] HU, while for ROI_enhancement_, the threshold was [c, d] HU. Then, we adjusted the threshold of the ROI_low density_ as [a, (b + c)/2)] and defined it as ROI_lesion_. The ROI_lesion_ was regionally segmented, and the lesion area was then automatically measured using a computer to obtain the volumes of hepatic lesions. We also calculated the ratio of ROI_lesion_ volume to ROI_liver_ volume (Ratio). The ROI_liver_ and ROI_lesion_ of patients with PA-SOS were extracted by the threshold-based region growing algorithm (Fig. 3).

### Statistical analysis

The statistical analysis was performed using SPSS version 17.0 (SPSS Inc., Chicago, IL, USA). The volume measurements of the ROI _liver_, ROI _lesion_ and Ratio were compared against clinical severity and patient outcomes and analyzed by one-way analysis of variance (ANOVA). An LSD test was used to compare intergroup variables. The volume measurement of the Ratio was correlated with liver function test parameters and analyzed with the Pearson correlation test. A P value less than 0.05 was considered to indicate a significant difference.

### Ethical approval and informed consent

Institutional Review Board approval was obtained by ethics committee of Anhui Medical University. Approval for this retrospective study was obtained from the ethics committee, and the requirement for informed consent was waived (approval number: 20170219).

## Data Availability

The datasets generated and analyzed during the current study are available from the corresponding author on reasonable request.
